# Proliferative sparganosis caused by *Spirometra* sp. 3 in a dog

**DOI:** 10.1177/10406387261438437

**Published:** 2026-04-22

**Authors:** Lillian R. Miller, Sharon K. Harley, Isel L. Pollock, Hayley K. Amerman, Caroline Sobotyk

**Affiliations:** Department of Pathobiology, University of Pennsylvania School of Veterinary Medicine, Philadelphia, PA, USA; North Saucon Animal Hospital, Bethlehem, PA, USA; Department of Pathobiology, University of Pennsylvania School of Veterinary Medicine, Philadelphia, PA, USA; Department of Pathobiology, University of Pennsylvania School of Veterinary Medicine, Philadelphia, PA, USA; Department of Pathobiology, University of Pennsylvania School of Veterinary Medicine, Philadelphia, PA, USA

**Keywords:** canine sparganosis, cestodiasis, companion animals, diphyllobothriidea, *Sparganum proliferum*, *Spirometra* tapeworms, zoonosis

## Abstract

Here, we report a case of proliferative sparganosis caused by *Spirometra* sp. 3 infection in a domestic dog in Pennsylvania, USA. Proliferative sparganosis is an unusual and often fatal condition caused by the multiplication of the larval cestode stage, *Spirometra* spp., within various organs and tissues of intermediate hosts, including humans, dogs, and cats. Although domestic dogs and cats are often definitive hosts of *Spirometra* spp., they can also act as paratenic or second intermediate hosts. These animals may develop high parasite burdens within muscle, connective tissue, or visceral organs after ingesting the cestode plerocercoid larvae through predation, scavenging, or contaminated water or food. As a zoonotic agent with potentially devastating consequences, accurate species identification through molecular and phylogenetic analysis is imperative for a better understanding of the *Spirometra* genus and other poorly understood parasites.

Proliferative sparganosis is a rare condition that results from extensive tissue infection by reproductively capable tapeworm larvae in the genus *Spirometra.* Disseminated *Spirometra* infection in paratenic or intermediate hosts by plerocercoid or third-stage larvae (spargana) has been reported in humans, dogs, cats, apes, and pigs.^2,4,7,8,12,18–20^ Typically, *non-proliferative infections*—in which only one or a few larvae migrate within the subcutis, musculature, or viscera—result in a mass effect with clinical implications largely restricted to mass location.^13^ The larvae form cystic spaces within a space-occupying mass. However, *proliferative sparganosis* develops from thousands of spargana that are capable of asexual reproduction. These larvae have a predilection to localize in dermal connective tissue but may be distributed within multiple cavities throughout the subcutis or viscera.^8,10,13,18^ Proliferative sparganosis is a distinct condition with greater morbidity and mortality than non-proliferative sparganosis. In humans, severe sparganosis infections have been reported to cause blindness, paralysis, and, in up to 30% of cases, death.^13,20^

Tapeworms of the genus *Spirometra* comprise a complex taxonomic group. Species in this genus are morphologically similar, and the historical data derived from poorly preserved or limited specimens have contributed to persistent misidentification affecting clinical outcomes, epidemiologic accuracy, and prevention and control strategies.^16^ With the implementation of molecular characterization, more precise speciation is possible. Three lineages—*Spirometra mansoni, Spirometra* sp. 2, and *Spirometra* sp. 3—have been confirmed in North America through molecular analysis.^16^ Historically, reports of proliferative sparganosis have been attributed to the species previously referred to as *Spirometra proliferum*.^4,12,18–20^ Here, we describe a novel case of proliferative sparganosis in a dog, with molecular confirmation of *Spirometra* sp. 3 as the causative agent.

Approximately 6 mo after adoption, a rescued adult, castrated male terrier-cross dog in Pennsylvania (originally adopted from Myrtle Beach, SC, USA) began vomiting. The patient was presented to a general veterinary practice in 2021; a biopsy taken during exploratory laparotomy revealed intraperitoneal cestodes. According to the managing veterinarian’s treatment plan, monthly praziquantel was administered for approximately one year following the initial procedure. The managing veterinarian utilized a veterinary consultation network, and the treatment dosage and frequency were chosen based on previous recommendations for a similar case of peritoneal cestodes. In 2024, a second laparotomy was performed to explore an inguinal mass. During the second laparotomy, free cestode larvae were noted within the peritoneal cavity. Larvae submitted to a veterinary diagnostic laboratory were morphologically identified as *Spirometra* sp. The patient received 2 consecutive doses of praziquantel at 25 mg/kg. During post-operative recovery, poor wound healing was noted as larvae were continually extruded through the incision site. Euthanasia was elected.

On postmortem examination, a draining tract in the right inguinal region communicated with an ~5-cm subcutaneous cavity. Within the cavity were numerous thin, white-to-tan, ribbon-like, 0.5–5.5-cm, cestode larvae (**Fig. 1A**). The abdominal muscles and peritoneum were thickened by abundant tan-to-red fibrous connective tissue, and within the thickened peritoneum were several regions of soft, friable tissue that encased cestode larvae. Deep to the thickened peritoneum, all abdominal viscera were encased within a thick layer of fibrous tissue, and free cestode larvae were found between the peritoneum and the layer of encapsulating fibrosis (**Fig. 1B**). The cestode larvae were collected and stored in 70% ethanol for molecular analysis. Samples of heart, lung, liver, kidney, small intestine, and abdominal wall were collected and fixed in 10% buffered, pH 7.2, formalin before being processed routinely to produce 4-µm-thick H&E-stained sections.

**Figure 1.**
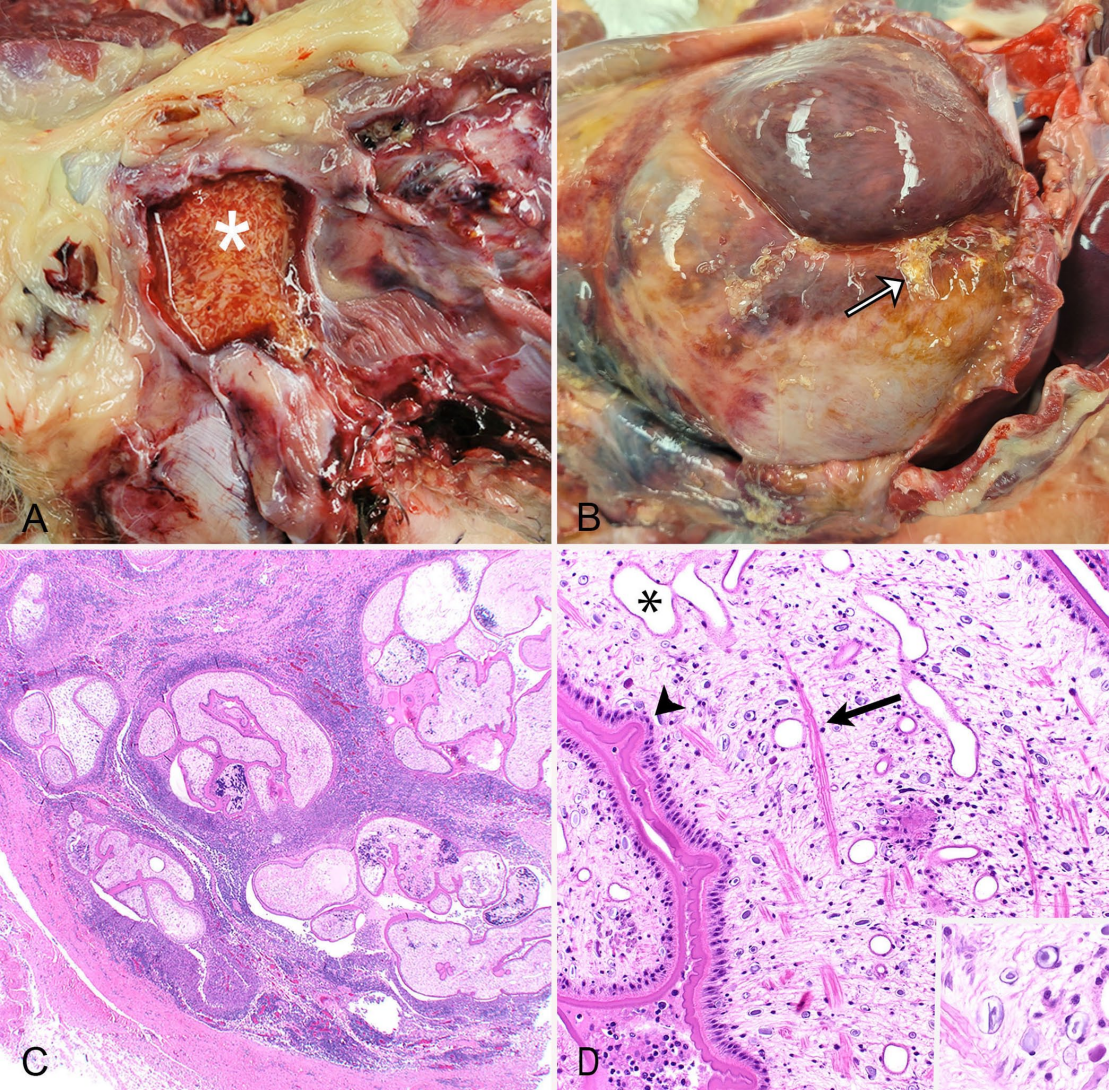
Proliferative sparganosis within the inguinal subcutis and peritoneum of a dog. **A.** The left inguinal adipose is expanded by a discrete cavity containing myriad plerocercoid larvae suspended in red, mucoid fluid (asterisk). **B.** Free plerocercoid larvae (arrow) within the peritoneal cavity. All viscera, except the liver, are encased in and obscured by a thick layer of fibrous tissue**. C.** The peritoneum is expanded by inflamed fibrous connective tissue. Cystic spaces containing cross-sections of plerocercoid larvae are interspersed within the fibrous stroma. H&E. **D.** Higher magnification of an individual larva with multiple invaginations (arrowhead) of the tegument. Within the parenchymal body are excretory ducts (asterisk) and muscle bundles (arrow). Inset: higher magnification of calcareous corpuscles. H&E.

Histologically, the peritoneum was expanded by bands of mature fibrous connective tissue that were heavily infiltrated by epithelioid macrophages, lymphocytes, and plasma cells. Interspersed within the fibrous connective tissue were many cystic spaces that contained several cross sections of unsegmented plerocercoid cestode larvae. Larvae were 0.25–0.5-mm diameter and were often surrounded by a rim of epithelioid macrophages (**Fig. 1C**). The larvae had solid parenchymal bodies with a thick, eosinophilic, folded tegument. Scattered within the bodies of the cestode larvae were basophilic bodies with concentric rings (calcareous corpuscles), muscle fibers, and excretory ducts (**Fig. 1D**). The larvae lacked scolices, suckers, and internal reproductive or gastrointestinal tracts. These morphologic features were consistent with *Spirometra* plerocercoid larvae.

Defining features of cestodes include multiple segments of solid parenchyma that are surrounded by a thick, eosinophilic tegument. Within the *Spirometra* genus, the tegument is often invaginated.^13^ Unlike trematodes, cestode larvae lack developed gastrointestinal and reproductive tracts and embedded within their parenchyma are calcareous corpuscles.^3^ The identification of specific morphologic features in the anterior portion can further categorize cestode larvae.^15^ Cyclophyllidean tapeworms (which include the families *Taeniidae*, *Dilepididae*, and *Mesocestoididae*) commonly infect dogs and cats, and pseudophyllidean tapeworms (which include *Spirometra* spp.) are the most common groups identified histologically.^5,17^ A lack of scolices or suckers is indicative of pseudophyllidean plerocercoid larvae; identification of suckers and scolices supports cyclophyllidean lineage. Multiple sections should be examined to ensure these features are not within deeper sections before identifying pseudophyllidean larvae.^5,15^

Genomic DNA was extracted from a fragment of a 70% ethanol-preserved *Spirometra* plerocercoid (DNeasy blood & tissue kit; Qiagen) according to the manufacturer’s instructions. We amplified the partial cytochrome oxidase c subunit 1 (*cox1*) region of mitochondrial DNA using the forward primer PlatCOI F (5′-TTTTTTGGGCATCCTGAGGTTTAT-3′) and reverse primer PlatCOI R (5′-TAAAGAAAGAACATAATGAAAATG-3′).^1^ Cycling conditions included denaturation at 95°C for 2 min, followed by 35 cycles of 95°C for 30 s, 52°C for 1 min, and 72°C for 1 min, followed by a final extension at 72°C for 5 min. Nuclease-free water was used as a negative control, and a fragment of a 70% ethanol-preserved *Dipylidium caninum* proglottid was used as the positive control. The PCR products were purified (E.Z.N.A. cycle pure kit; Omega Bio-tek), then sequenced in both directions using the original PCR primers via Sanger sequencing (3730XL DNA analyzer; Penn Genomics and Sequencing core, Philadelphia, PA, USA).

Phylogenetic analysis was performed using the maximum-likelihood method with 1,000 bootstrap support and the Tamura–Nei model with gamma-distribution (TN93+G) best-fit substitution model in MEGA X v.10.2.^11^ *Schistocephalus solidus* (GenBank AP017669) was used as an outgroup. The generated 419-bp *cox1* sequence (GenBank PX392491) had 98.5–99.7% maximum identity with *Spirometra* sp. 3 sequences in GenBank. Subsequent phylogenetic analysis confirmed that our isolate has a close genetic relationship with other *Spirometra* sp. 3 isolates from the United States (**Fig. 2**).

**Figure 2.**
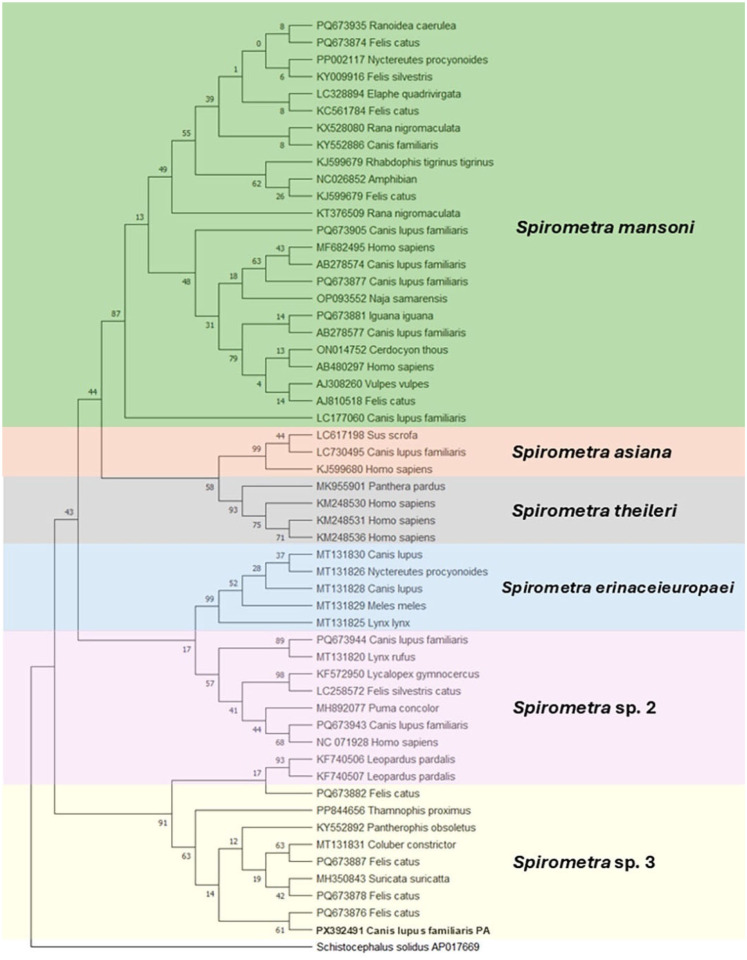
Phylogenetic relationship based on partial cytochrome oxidase c subunit 1 gene of *Spirometra* sp. 3 plerocercoid specimen collected from a domestic dog in Pennsylvania, USA (GenBank PX392491) and other *Spirometra* spp. sequences in GenBank. The colors highlight the well-defined, distinct *Spirometra* lineages. Analysis was by the maximum-likelihood method (1,000 bootstrap replicates) in MEGA X. The best substitution model used was the Tamura–Nei with a discrete gamma distribution. Branches corresponding to partitions reproduced in <50% bootstrap replicates were collapsed. *Schistocephalus solidus* was used as an outgroup.

Tapeworms within the genus *Spirometra* are well-known parasites of wildlife, commonly using carnivorous mammals as definitive hosts.^10,16^ To complete its lifecycle, *Spirometra* requires 2 intermediate hosts. After contacting freshwater, a cestode ovum releases the first larval stage, known as a coracidium. A copepod acts as the first intermediate host and consumes the coracidium.^16^ In the copepod, the lifecycle continues with development into a procercoid larva. A second intermediate host ingests the infected copepod, and the larva then develops into the plerocercoid stage, also known as a sparganum. Second intermediate hosts often include amphibians, reptiles, mammals, and, less commonly, birds.^8,16,18–20^ The intermediate hosts containing spargana are consumed by definitive hosts, allowing completion of the lifecycle through fecal shedding of ova from the definitive host. Dogs and cats are the most common definitive hosts, but adult *Spirometra* has been reported in at least 67 different mammals.^10,16^

When infected with adult *Spirometra*, clinical signs, when present, are usually limited to vomiting, diarrhea, and weight loss.^16^ However, if a paratenic host ingests the plerocercoid larva (sparganum), migration occurs through the subcutaneous and visceral tissue, including the liver, lungs, and brain.^7, 8^ Dogs, cats, and humans can act as definitive, intermediate, or paratenic hosts.^8,18,19^ Humans typically become infected after consuming contaminated drinking water, and the subsequent larval migration can result in local damage, ocular or neurologic disease, and death.^12,13,16^ Infections are usually non-proliferative, with only a few larvae migrating through tissue. However, in some cases, a few plerocercoid lineages may undergo asexual reproduction, resulting in proliferative sparganosis with numerous invasive organisms.^2,4,8,12,13,18^ All tissues may be affected, and the infection ultimately results in the death of the host.^20^

In our case, the consumption of an infected copepod was likely the source of this dog’s proliferative larval cestode infection. Given that the peritoneal plerocercoid larvae were discovered within 6 mo of adoption, exposure to procercoid-containing copepods could have occurred from freshwater sources in either South Carolina or Pennsylvania. The North American *Spirometra* sp. 3 lineage has been reported in both states in domestic and exotic animals.^16^ An inherent risk of translocating domestic animals within the United States is the movement of zoonotic pathogens and disease transmission. Our case of proliferative sparganosis underscores the importance of targeted educational strategies, disease surveillance, and effective prevention measures as means of controlling devastating zoonoses.

The ability of dogs and cats to serve as both definitive and intermediate hosts of *Spirometra* spp. contributes to environmental contamination, parasite maintenance, and transmission. Given their close proximity to humans, domestic animals may act as amplifier hosts, enhancing contamination of freshwater environments, and increasing risk of human exposure through the ingestion of infected copepods or via larval entry through open wounds.^10,13^ Prevention of proliferative sparganosis, the most severe clinical manifestation of *Spirometra* infection, should focus on limiting exposure and preventing transmission, given that no FDA-approved drugs or consistently effective treatments are available for companion animals.^3,4^ In human medicine, a standard treatment protocol has been difficult to establish and no consensus exists regarding the optimal therapy. In a limited number of small retrospective studies, long-term, high-dose praziquantel has been reported as an alternative to traditional surgical larval removal in cases of cerebral sparganosis in humans.^6,21^ These findings offer a potentially less-invasive treatment option for deeply embedded or proliferative sparganosis infections in animals and offers a possible rationale for the treatment strategy used in our case. To prevent initial infection in highly contaminated areas, filtering water may reduce exposure to infected copepods. Expanding awareness of this neglected zoonotic disease and educating the public on how to avoid infection is vital given that medical and surgical therapies are often unsuccessful in treating proliferative sparganosis.^16^

Although its taxonomy is still being refined, the genus *Spirometra* has been reclassified into 7 lineages defined by geographic location and genetic diversity. In North America, 3 different lineages are present, including *S. mansoni* and *Spirometra* sp. 2 and 3 (previously known as *S. decipiens* complex 1 and 2).^10,16^ Cases of proliferative sparganosis have been associated with *Spirometra* sp. 2 in animals and humans in the Americas and *S. mansoni* in a cat in Asia.^8,9,19^ In comparison, *Spirometra* sp. 3 has been associated with subcutaneous sparganosis in reptiles and meerkats, and intestinal infections in domestic cats only.^14,16^ To our knowledge, *Spirometra* sp. 3 has not been reported previously as a cause of proliferative sparganosis in a domestic dog. We retrieved no cases documenting proliferative sparganosis with *Spirometra* sp. 3 in dogs in a search of Google, PubMed, CAB Direct, Web of Science, and Scopus, using the search terms “dog”, “proliferative sparganosis”, and “*Spirometra* sp. 3”.

Our findings highlight the need for molecular analysis and characterization for accurate diagnosis, particularly for internal proliferative sparganosis cases. Molecular methods and phylogenetic analysis are crucial for correct parasite identification, improving our understanding of parasite biology, distribution, and epidemiology, and clarifying the clinical relevance and transmission risks of the different *Spirometra* lineages for both animals and humans.
